# A Hybrid Common Fixed Point Theorem under Certain Recent Properties

**DOI:** 10.1155/2014/860436

**Published:** 2014-01-23

**Authors:** Zoran Kadelburg, Sunny Chauhan, Mohammad Imdad

**Affiliations:** ^1^Faculty of Mathematics, University of Belgrade, Studentski Trg 16, 11000 Beograd, Serbia; ^2^R. H. Government Postgraduate College, Kashipur, Uttarakhand 244713, India; ^3^Department of Mathematics, Aligarh Muslim University, Aligarh 202 002, India

## Abstract

We prove a common fixed point theorem for a hybrid pair of occasionally coincidentally idempotent mappings via common limit range property. Our result improves some results from the existing literature, especially the ones contained in Sintunavarat and Kumam (2009). Some illustrative and interesting examples to highlight the realized improvements are also furnished.

## 1. Introduction and Preliminaries

Nadler's contraction principle [1] is the generalization of the classical Banach contraction principle to the case of multivalued mappings. Hybrid fixed point theory for single-valued and multivalued mappings is a new development in nonlinear analysis (see, e.g., [2–7] and references therein). The concepts of commutativity and weak commutativity were extended to multivalued mappings on metric spaces by Kaneko [8, 9]. In 1989, Singh et al. [10] extended the notion of compatible mappings and obtained some coincidence and common fixed point theorems for nonlinear hybrid contractions. It was observed that under compatibility the fixed point results always require continuity of one of the underlying mappings. Afterwards, Pathak [11] generalized the concept of compatibility by defining weak compatibility for hybrid pairs of mappings (including single-valued case) and utilized the same to prove common fixed point theorems. Naturally, compatible mappings are weakly compatible but not conversely. For an extensive collection of hybrid contraction conditions, we refer to [12–21].

The following definitions and results will be needed in the sequel.

Let (*X*, *d*) be a metric space. Then, on the lines of Nadler [1], we adopt that

(1) *CL*(*X*) = {*A* : *A* is a nonempty closed subset of *X*},
(2) *CB*(*X*) = {*A* : *A* is a nonempty closed and bounded subset of *X*},
(3) for nonempty closed and bounded subsets *A*, *B* of *X* and *x* ∈ *X*
  (1) d(x,A)=inf⁡{d(x,a):a∈A},H(A,B)=max⁡{sup⁡{d(a,B):a∈A},     sup⁡{d(b,A):b∈B}}.

It is well known that *CB*(*X*) is a metric space with the distance *H* which is known as the Hausdorff-Pompeiu metric on *CB*(*X*).

The following terminology is also standard.

**Definition 1**

Let (*X*, *d*) be a metric space with *f* : *X* → *X* and *T* : *X* → *CB*(*X*).

1. A point *x* ∈ *X* is a fixed point of *f* (resp., *T*) if *x* = *fx* (resp., *x* ∈ *Tx*). The set of all fixed points of *f* (resp., *T*) is denoted by *F*(*f*) (resp., *F*(*T*)).
2. A point *x* ∈ *X* is a coincidence point of *f* and *T* if *fx* ∈ *Tx*. The set of all coincidence points of *f* and *T* is denoted by *C*(*f*, *T*).
3. A point *x* ∈ *X* is a common fixed point of *f* and *T* if *x* = *fx* ∈ *Tx*. The set of all common fixed points of *f* and *T* is denoted by *F*(*f*, *T*).

**Definition 2**

Let (*X*, *d*) be a metric space with *f* : *X* → *X* and *T* : *X* → *CB*(*X*). A hybrid pair of mappings (*f*, *T*) is said to be

1. commuting on *X* [8] if *f* *Tx*⊆*T* *fx* for all *x* ∈ *X*,
2. weakly commuting on *X* [9] if *H*(*f* *Tx*, *T* *fx*) ≤ *d*(*fx*, *Tx*) for all *x* ∈ *X*,
3. compatible [10] if *f* *Tx* ∈ *CB*(*X*) for all *x* ∈ *X* and lim⁡_*n*→*∞*_ *H*(*T* *fx*_*n*_, *f* *Tx*_*n*_) = 0, whenever {*x*_*n*_} is a sequence in *X* such that *Tx*_*n*_ → *A* ∈ *CB*(*X*) and *fx*_*n*_ → *t* ∈ *A*, as *n* → *∞*,
4. noncompatible [16] if there exists at least one sequence {*x*_*n*_} in *X* such that *Tx*_*n*_ → *A* ∈ *CB*(*X*) and *fx*_*n*_ → *t* ∈ *A*, as *n* → *∞*, but lim⁡_*n*→*∞*_ *H*(*T* *fx*_*n*_, *f* *Tx*_*n*_) is either nonzero or nonexistent,
5. weakly compatible [22] if *f* *Tx* = *T* *fx* for each *x* ∈ *C*(*f*, *T*),
6. occasionally weakly compatible [23] if *f* *Tx*⊆*T* *fx* for some *x* ∈ *C*(*f*, *T*),
7. coincidentally commuting [11] if they commute at their coincidence points,
8. coincidentally idempotent [14] if *ffv* = *fv* for every *v* ∈ *C*(*f*, *T*); that is, *f* is idempotent at the coincidence points of *f* and *T*,
9. occasionally coincidentally idempotent [24] if *ffv* = *fv* for some *v* ∈ *C*(*f*, *T*).

Now we present an example showing the relationship of the occasionally coincidentally idempotent notion to other conditions of the previous definition.

**Example 3**

Let *X* = {1,2, 3} (with the standard metric),

(2) $$\begin{array}{c} f:\left(\begin{array}{ccc} 1 & 2 & 3\\ 1 & 3 & 2 \end{array}\right),\;\;T:\left(\begin{array}{ccc} 1 & 2 & 3\\ \left\{1\right\} & \left\{1,3\right\} & \left\{1,3\right\} \end{array}\right). \end{array}$$

Then it is easy to see the following:

- *C*(*f*, *T*) = {1,2} since *f*1 = 1 ∈ {1} = *T*1, *f*2 = 3 ∈ {1,3} = *T*2, and *f*3 = 2 ∉ {1,3} = *T*3. Also, *F*(*f*, *T*) = {1}.
- (*f*, *T*) is not commuting and not weakly commuting since *H*(*fT*2, *Tf*2) = *H*({1,2}, {1,3}) = max⁡{1,1} = 1 > 0 = *d*(3, {1,3}) = *d*(*f*2, *T*2).
- (*f*, *T*) is not compatible since, for *x*_*n*_ = 2, *n* ∈ *ℕ*, we have that *Tx*_*n*_ = {1,3}→{1,3} = *A* and *fx*_*n*_ = 3 → 3 ∈ *A* as *n* → *∞*, but *H*(*f* *Tx*_*n*_, *T* *fx*_*n*_) = *H*({1,2}, {1,3}) = max⁡{1,1} = 1↛0 as *n* → *∞*.
- (*f*, *T*) is not weakly compatible since *fT*2 = {1,2}≠{1,3} = *Tf*2.
- (*f*, *T*) is not coincidentally commuting since *fT*2 = {1,2}⊈{1,3} = *Tf*2.
- (*f*, *T*) is not coincidentally idempotent since *ff*2 = *f*3 = 2 ≠ 3 = *f*2.
- (*f*, *T*) is occasionally coincidentally idempotent since *ff*1 = 1 = *f*1.

Obviously, in this case (*f*, *T*) is also noncompatible and occasionally weakly compatible, but simple modification of this example shows that the occasionally coincidentally idempotent property is independent of these two notions. For example, if

(3) $$\begin{array}{c} f:\left(\begin{array}{ccc} 1 & 2 & 3\\ 1 & 3 & 2 \end{array}\right),\;\;T:\left(\begin{array}{ccc} 1 & 2 & 3\\ \left\{1,2\right\} & \left\{1,2\right\} & \left\{1,3\right\} \end{array}\right), \end{array}$$

then the pair (*f*, *T*) is occasionally coincidentally idempotent but not occasionally weakly compatible.

**Remark 4**

It was shown in [25] that, in the case of single-valued mappings, the occasionally weakly compatible property does not produce new common fixed point results, since it reduces to weak compatibility in the presence of a unique point of coincidence. However, [25, Example 2.5] shows that an analogue conclusion does not hold in the case of hybrid pairs. Hence, in this case, the notion of occasionally weak compatibility might still produce new results. A similar example can be presented, showing the possibility of usage of the occasionally coincidentally idempotent notion.

Inspired by the work of Aamri and Moutawakil [26], Kamran [27] extended the notion of property (E.A) for a hybrid pair of mappings.

**Definition 5**

Let (*X*, *d*) be a metric space with *f* : *X* → *X* and *T* : *X* → *CB*(*X*). The hybrid pair of mappings (*f*, *T*) is said to satisfy the property (E.A) if there exists a sequence {*x*_*n*_} in *X* such that

(4) $$\begin{array}{c} \underset{n\to \infty }{\lim}fx_{n}=t\in A=\underset{n\to \infty }{\lim}Tx_{n}, \end{array}$$

for some *t* ∈ *X* and *A* ∈ *CB*(*X*).

In 2011, Sintunavarat and Kumam [28] introduced the notion of common limit range property for single-valued mappings and showed its superiority over property (E.A). Motivated by this fact, Imdad et al. [29] established common limit range property for a hybrid pair of mappings and proved some fixed point results in symmetric (semimetric) spaces.

**Definition 6 (see [29])**

Let (*X*, *d*) be a metric space with *f* : *X* → *X* and *T* : *X* → *CB*(*X*). Then the hybrid pair of mappings (*f*, *T*) is said to satisfy the common limit range property with respect to the mapping *f* if there exists a sequence {*x*_*n*_} in *X* such that

(5) $$\begin{array}{c} \underset{n\to \infty }{\lim}fx_{n}=fu\in A=\underset{n\to \infty }{\lim}Tx_{n}, \end{array}$$

for some *u* ∈ *X* and *A* ∈ *CB*(*X*).

Now, we present some examples demonstrating the preceding definition.

**Example 7**

Let us consider *X* = [0,2] with the usual metric *d*(*x*, *y*) = |*x* − *y*|. Define *f* : *X* → *X* and *T* : *X* → *CB*(*X*) as follows:

(6) $$\begin{array}{c} fx=\{\begin{array}{cc} 2-x, & \text{if}\;\;0\leq x<1,\\ \frac{9}{5}, & \text{if}\;\;1\leq x\leq 2, \end{array}\;\\ Tx=\{\begin{array}{cc} \left[\frac{1}{2},\frac{3}{2}\right], & \text{if}\;\;0\leq \;x\leq 1,\\ \left[\frac{1}{4},\frac{1}{2}\right], & \text{if}\;\;1<x\leq 2.\; \end{array} \end{array}$$

One can verify that the pair (*f*, *T*) enjoys the property (E.A) as, considering the sequence {*x*_*n*_} = {1−1/*n*}_*n*∈*ℕ*_, one gets that

(7) $$\begin{array}{c} \underset{n\to \infty }{\lim}fx_{n}=1\left(=t\right)\in \left[\frac{1}{2},\frac{3}{2}\right]=\underset{n\to \infty }{\lim}Tx_{n}. \end{array}$$

However, there exist no *u* in *X* for which *t* = *fu*.

**Example 8**

In the setting of Example 7, replace the mapping *f* by the following, besides retaining the rest:

(8) $$\begin{array}{c} fx=\{\begin{array}{cc} 2-x, & \text{if}\;\;0\leq x\leq 1,\\ \;\frac{9}{5}, & \text{if}\;\;1<x\leq 2. \end{array} \end{array}$$

Then the pair (*f*, *T*) satisfies the common limit range property with respect to the mapping *f* (for the sequence {*x*_*n*_} = {1−1/*n*}_*n*∈*ℕ*_) as

(9) $$\begin{array}{c} \underset{n\to \infty }{\lim}fx_{n}=1=f\left(1\right)\in \left[\frac{1}{2},\frac{3}{2}\right]=\underset{n\to \infty }{\lim}Tx_{n}. \end{array}$$

**Remark 9**

Note that, if a pair (*f*, *T*) satisfies the property (E.A) along with the closedness of *f*(*X*), then the pair also satisfies the common limit range property with respect to the mapping *f*.

The aim of this note is to prove a common fixed point theorem for a hybrid pair of mappings by using the notion of common limit range property (due to Imdad et al. [29]) along with occasionally coincidentally idempotent property (due to Pathak and Rodriguez-Lopez [24]).

## 2. Main Result

We first state the following theorem due to Sintunavarat and Kumam [30] proved for a pair of hybrid pair of mappings by using the property (E.A).

**Theorem 10 (see [30, Theorem 3.1])**

Let *f* be a self mapping of a metric space (*X*, *d*) and let *T* be a mapping from *X* into *CB*(*X*) such that the following conditions are satisfied:

(1) *f* and *T* satisfy the property (E.A),
(2) for all *x*, *y* ∈ *X* with *x* ≠ *y*,
  (10) H(Tx,Ty) ≤max⁡{d(fx,fy),12[d(fx,Tx)+d(fy,Ty)],      12[d(fx,Ty)+d(fy,Tx)]},
(3) *ffv* = *fv* for all *v* ∈ *C*(*f*, *T*) (i.e., (*f*, *T*) is coincidentally idempotent).

If *f*(*X*) is a closed subset of *X*, then *f* and *T* have a common fixed point.

Now we utilize the occasionally coincidetally idempotent notion (which is weaker than coincidentally idempotent one in the case when the set of coincidence points is not empty; see Example 3) and common limit range property (instead of property (E.A)) and prove a respective result without any requirement of closedness of the range of *f*.

**Theorem 11**

Let *f* be a self mapping of a metric space (*X*, *d*) and let *T* be a mapping from *X* into *CB*(*X*) satisfying condition (10) of Theorem 10. Suppose that the pair (*f*, *T*) enjoys the common limit range property with respect to the mapping *f*. Then the mappings *f* and *T* have a coincidence point (i.e., *C*(*f*, *T*) ≠ *∅*).

Moreover, if the pair (*f*, *T*) enjoys occasionally coincidentally idempotent property (i.e., *ffv* = *fv*  for some *v* ∈ *C*(*f*, *T*)), then the pair (*f*, *T*) has a common fixed point.

**Proof**

Suppose that the pair (*f*, *T*) satisfies the common limit range property with respect to the mapping *f*; then there exists a sequence {*x*_*n*_} in *X* such that

(11) $$\begin{array}{c} \underset{n\to \infty }{\lim}fx_{n}=fu\in A=\underset{n\to \infty }{\lim}Tx_{n}, \end{array}$$

for some *u* ∈ *X* and *A* ∈ *CB*(*X*). We assert that *fu* ∈ *Tu*. If not, then, using condition (10), we get

(12) H(Txn,Tu) ≤max⁡{d(fxn,fu),12[d(fxn,Txn)+d(fu,Tu)],     12[d(fxn,Tu)+d(fu,Txn)]}.

Taking the limit as *n* → *∞*, we have

(13) H(A,Tu)≤max⁡{0,12[d(fu,A)+d(fu,Tu)], 12[d(fu,Tu)+d(fu,A)]}.

Since *fu* ∈ *A*, the above inequality implies that

(14) d(fu,Tu)≤H(A,Tu)≤max⁡{12d(fu,Tu),12d(fu,Tu)},=12d(fu,Tu),

which is a contradiction. Hence *fu* ∈ *Tu* which shows that the pair (*f*, *T*) has a coincidence point (i.e., *C*(*f*, *T*) ≠ *∅*).

If the mappings *f* and *T* are occasionally coincidentally idempotent, two cases arise: *f* and *T* may be or may not be coincidentally idempotent at *u*.

*Case I.* If *f* and *T* are coincidentally idempotent at *u*, then we have *ff* *u* = *fu* ∈ *Tu*. Now we show that *Tu* = *Tf* *u*. If not, using condition (10), we get

(15) H(Tfu,Tu) ≤max⁡{d(ffu,fu),12[d(ffu,Tfu)+d(fu,Tu)],     12[d(ffu,Tu)+d(fu,Tfu)]} =max⁡{0,12[d(fu,Tfu)+d(fu,Tu)],     12[d(fu,Tu)+d(fu,Tfu)]}.

Since *fu* ∈ *Tu*, the above inequality implies that

(16) d(Tfu,fu)≤H(Tfu,Tu)≤max⁡{12d(fu,Tfu),12d(fu,Tfu)}=12d(Tfu,fu),

which is a contradiction. Thus we have *fu* = *ff* *u* ∈ *Tu* = *Tf* *u* which shows that *fu* is a common fixed point of the mappings *f* and *T*.

*Case II.* If *f* and *T* are not coincidentally idempotent at *u*, then, by the virtue of occasionally coincidentally idempotent property of *f* and *T*, there exists a coincidence point *u*′ ∈ *X* of *f* and *T* at which *f* and *T* are coincidentally idempotent; that is, *ff* *u*′ = *fu*′. The rest of the proof of this case can be completed on the similar lines as has been done in Case I when *f* and *T* are coincidentally idempotent at *u*.

**Example 12**

Let *X* = [0, +*∞*) be equipped with the standard metric. Consider the mappings *f* : *X* → *X* and *T* : *X* → *CB*(*X*) given by

(17) $$\begin{array}{c} fx=\{\begin{array}{cc} x^{2}, & 0\leq x<1,\\ \frac{1}{2}\left(x^{2}+x+1\right), & 1\leq x<2,\\ x^{2}-\frac{3}{2}, & x\geq 2, \end{array}\\ Tx=\left[0,\frac{1}{2}\left(x^{2}+1\right)\right]. \end{array}$$

Then,

- *f*(*X*) = [0,1)∪[3/2, +*∞*) is not closed in *X*;
- *C*(*f*, *T*) = [0,1)∪{2};
- for *x*_*n*_ = 1/*n*, lim⁡_*n*→*∞*_ *fx*_*n*_ = 0 = *f*0 ∈ {0} = *T*0 = lim⁡_*n*→*∞*_ *Tx*_*n*_; hence, (*f*, *T*) enjoys the common limit range property with respect to the mapping *f*;
- *ff*0 = *f*0 = 0; hence, (*f*, *T*) is occasionally coincidentally idempotent;
- *ff*2 = *f*(5/2) = 19/4 ≠ *f*2; hence, (*f*, *T*) is not coincidentally idempotent;
- *fT*2 = *f*([0,5/2]) = [0,1)∪[3/2,19/4]⊈[0,29/8] = *T*(5/2) = *Tf*2; hence, (*f*, *T*) is not coincidentally commuting (and not weakly compatible).

In order to check the contractive condition (10) of Theorem 11, without loss of generality, we can suppose that 0 ≤ *x* < *y* < *∞*. Then *H*(*Tx*, *Ty*) = (1/2)(*y* ^2^ − *x* ^2^). Consider the following possible cases:

(1) 0 ≤ *x* < *y* < 1; then *d*(*fx*, *fy*) = *y* ^2^ − *x* ^2^ ≥ *H*(*Tx*, *Ty*);
(2) 1 ≤ *x* < *y* < 2; then
  (18) d(fx,fy)=12(y2+y−x2−x)≥12x+yx+y+1(y−x)(y+x+1)=12(y2−x2)=H(Tx,Ty);
(3) 2 ≤ *x* < *y*; then *d*(*fx*, *fy*) = *y* ^2^ − *x* ^2^ ≥ *H*(*Tx*, *Ty*);
(4) 0 ≤ *x* < 1 ≤ *y* < 2; then
  (19) 12[d(x,fy)+d(y,fx)]=12[12(y2+y+1)−x+y−x2]≥12(y2−x2)=H(Tx,Ty),
  since (1/2)(*y* ^2^ + *y* + 1) − *x* + *y* − *x* ^2^ − (*y* ^2^ − *x* ^2^) = (1/2)(−*y* ^2^ + 3*y* + 1) − *x* ≥ 3/2 − 1 = 1/2 > 0;
(5) 0 ≤ *x* < 1, *y* ≥ 2; then
  (20) d(fx,fy)=y2−32−x2≥52−x2>12(x2−4)≥12(x2−y2)=H(Tx,Ty);
(6) 1 ≤ *x* < 2 ≤ *y*; this case is similar to case (2).

Hence, all the conditions of Theorem 11 are fulfilled and the pair (*f*, *T*) has a common fixed point (which is 0). The same conclusion cannot be obtained using Theorem 10 since *f*(*X*) is not closed and (*f*, *T*) is not coincidentally idempotent.

In view of Remark 9, we have the following natural result which still improves the results of Sintunavarat and Kumam [30] as the notion of occasionally coincidentally idempotent is more general than coincidentally idempotent.

**Corollary 13**

Let *f* be a self mapping of a metric space (*X*, *d*) and let *T* be a mapping from *X* into *CB*(*X*) satisfying condition (10) of Theorem 10. Suppose that the pair (*f*, *T*) satisfies the property (E.A) along with the closedness of *f*(*X*). Then the mappings *f* and *T* have a coincidence point (i.e., *C*(*f*, *T*) ≠ *∅*).

Moreover, if the pair (*f*, *T*) enjoys occasionally coincidentally idempotent property (i.e., *ffv* = *fv* for some *v* ∈ *C*(*f*, *T*)), then the pair (*f*, *T*) has a common fixed point.

Notice that a noncompatible hybrid pair always satisfies the property (E.A). Hence, we get the following corollary.

**Corollary 14**

Let *f* be a self mapping of a metric space (*X*, *d*) and let *T* be a mapping from *X* into *CB*(*X*) satisfying condition (10) of Theorem 10. Suppose that the pair (*f*, *T*) is noncompatible and *f*(*X*) is a closed subset of *X*. Then the mappings *f* and *T* have a coincidence point (i.e., *C*(*f*, *T*) ≠ *∅*).

Moreover, if the pair (*f*, *T*) enjoys occasionally coincidentally idempotent property (i.e., *ffv* = *fv* for some *v* ∈ *C*(*f*, *T*)), then the pair (*f*, *T*) has a common fixed point.
